# Electrical Conductivity Distribution in Detonating Benzotrifuroxane

**DOI:** 10.1038/s41598-018-28028-2

**Published:** 2018-06-25

**Authors:** Nataliya Satonkina, Alexander Ershov, Alexey Kashkarov, Anatoly Mikhaylov, Eduard Pruuel, Ivan Rubtsov, Ivan Spirin, Victoria Titova

**Affiliations:** 1 Lavrentyev Institute of Hydrodynamics SB RAS, pr. ac. Lavrentyeva, 15, Novosibirsk, 630090 Russia; 2 Novosibirsk State University, Pirogova str., 1, Novosibirsk, 630090 Russia; 3 Russian Federal Nuclear Center - VNIIEF, Sarov, Nizhny Novgorod region, Muzrukov Ave, 10, 607188 Russia

## Abstract

Electrical conductivity profile behind the detonation front in the benzotrifuroxane (BTF) was measured using high-resolution technique. BTF is a peculiar high explosive which is completely hydrogen-free: its molecular formula is C_6_N_6_O_6_. Results are compared with the conductivity distributions in detonating hexogen (RDX, C_3_H_6_N_6_O_6_) and triaminotrinitrobenzene based explosive (TATB, C_6_H_6_N_6_O_6_). The conductivity in BTF was found to be similar to that observed in the common explosives which contain hydrogen. Thus, the contribution of hydrogen (e.g., ions produced by the dissociation of water) in the conductivity is minor, both in the reaction zone and in the final detonation products. The characteristics of the conductivity profiles generally support the idea of contact conductivity through the connected structures of carbon particles formed in the detonation wave.

## Introduction

Benzotrifuroxane is a powerful high explosive with several important features which might affect the electrical conductivity of the detonating explosive. Thus, a study of the conductivity distribution in the detonation of BTF is of certain interest.

One possible mechanism of rather high electrical conductivity in explosion was suggested by Dremin,Yakushev and Antipenko^1,2^. They pointed out that the most typical detonation product is water. Dissociation of water enhanced by the extreme parameters of detonation might produce ions which are regarded as main charge carriers. BTF molecule does not contain hydrogen, therefore water is absent in the detonation products. According to the ionic hypothesis, the conductivity in BTF should be essentially different from that in more common explosives which contain hydrogen.

A number of thermodynamic calculations report quite high detonation temperature in BTF (4900 K^3^, 5700 K^4^). However, recent measurements^5^ demonstrated a lower value (about 4100 K). Given the significant discrepancy between the theory and the experiments, the actual temperature may have some intermediate value, higher than that measured in other explosives, which might affect the conductivity distribution.

**Figure 1:**
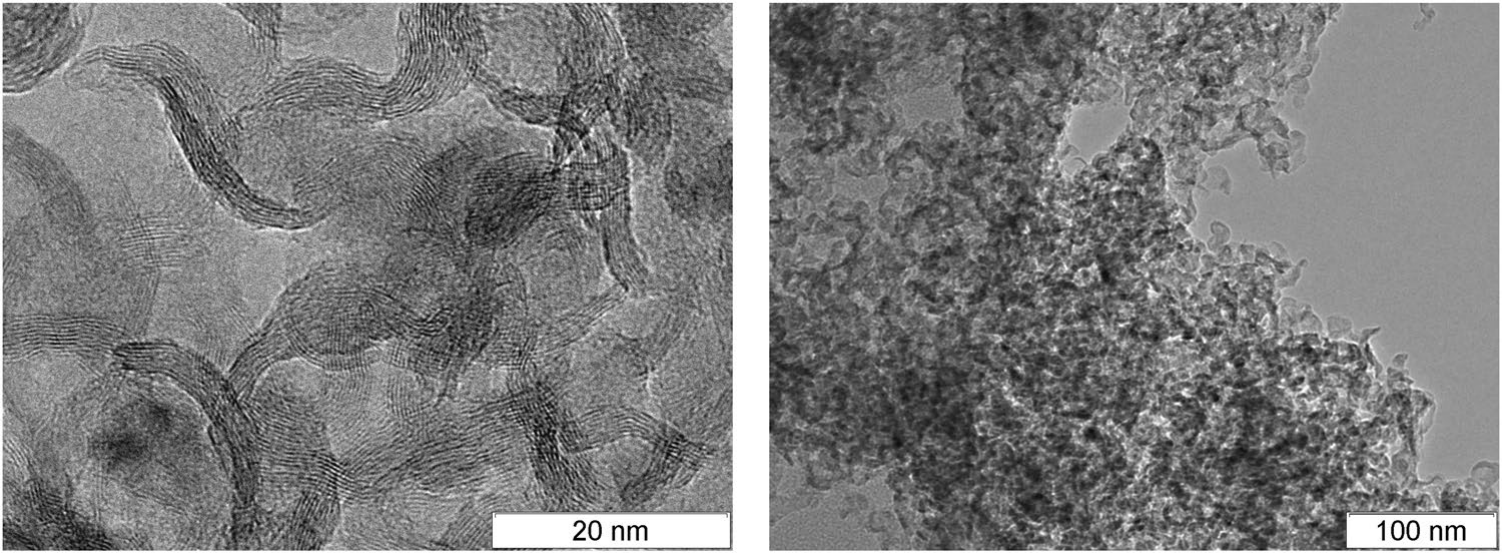
Carbon residuum of TATB detonation.

**Figure 2:**
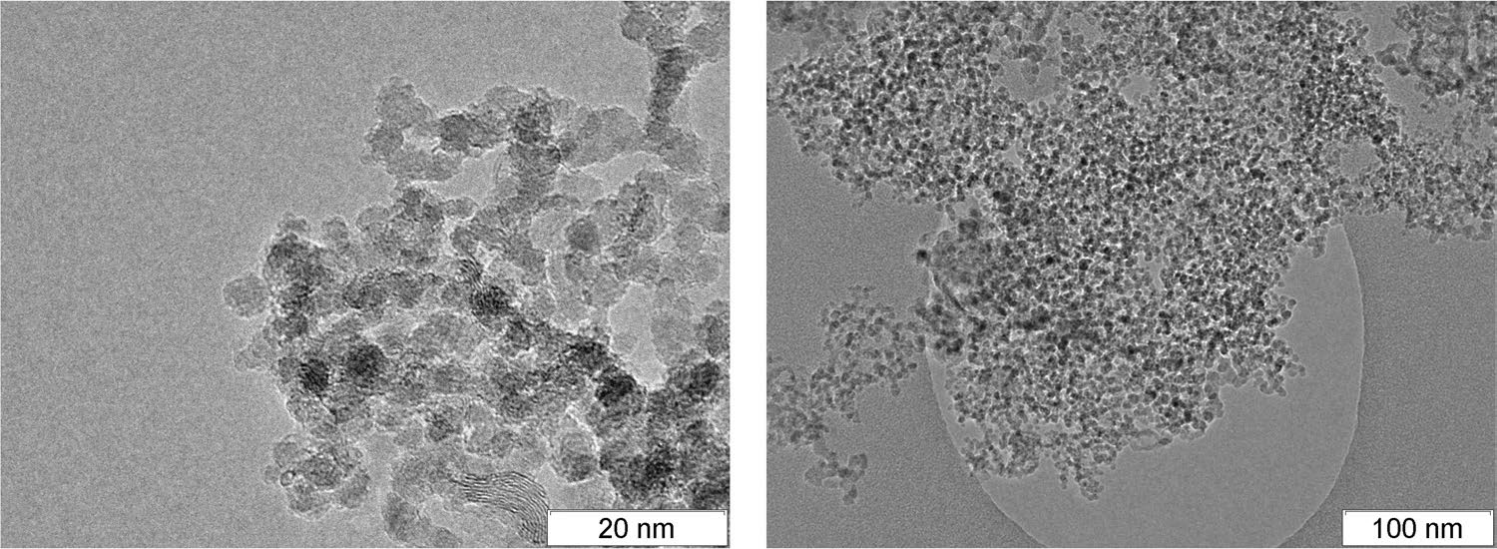
Carbon residuum of TNT/RDX 50/50 detonation.

**Figure 3:**
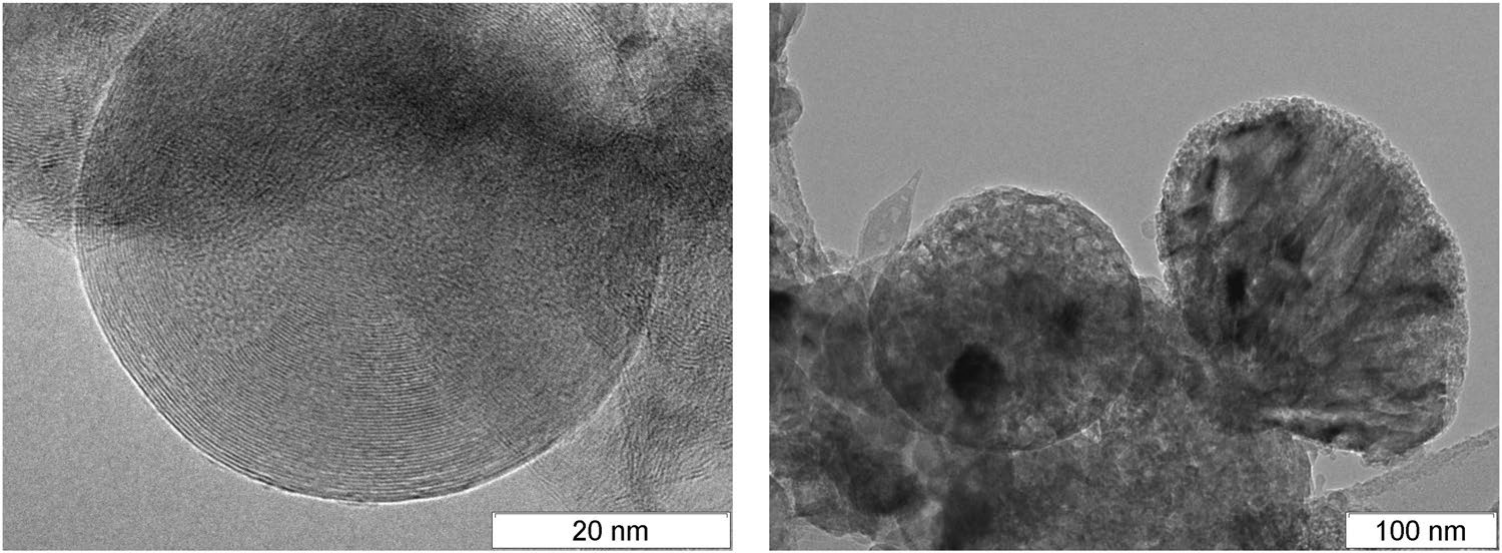
Carbon residuum of BTF detonation.

Finally, an important detonation product is solid carbon. A broad range of explosives was studied in this connection and many different structures were found (amorphous carbon, diamond, onion-like structures, filaments), with typical size of several nanometers^6–11^. The carbon particles formed in the detonation of BTF are much larger and they have rather complex inner structure. The carbon grains found in the detonation products of TATB, TNT/RDX 50/50 and BTF^12^ are compared in Figs 1, 2 and 3. The content of carbon in the source molecules of TATB and BTF is about the same, but the solid product differs both in size and in form. For BTF, the solid particles are large (from tens to hundreds of nanometers) and more or less spherical. For TATB, the characteristic diameter is under 20 nm, and one can see rather long fibers. Finally, for TNT/RDX mixture, the particles are still smaller, several nm in diameter, and mostly compact. A certain part of the solid product of TNT/RDX composition consists of the carbon which came from RDX molecules, so Fig. 2 is to some extent representative as illustration of RDX solid residuum. More electronic microscopy data can be found in http://ancient.hydro.nsc.ru/srexpl/detcarbon/index.html.

Authors of work^13^ suggested that the pecularities of the solid carbon produced by BTF are the consequence of high detonation temperature which may correspond to the liquid state at the phase diagram of carbon^14,15^. According to work^16^, large particles are formed owing to the absence of hydrogen. The conductivity profile might reflect these factors because the conductivity in the detonation wave depends strongly on the carbon content^17–20^.

In this paper, the measurements of electrical conductivity distribution in the detonating BTF are presented. BTF is a powerful explosive which contains 28.6 mass percent of carbon. The oxygen balance of BTF is −38.1%, while for TATB it is −55.8% and for RDX −21.6%. Results obtained with BTF are compared with those measured in other explosives.

## Elecrtical Conductivity Measurements

**Figure 4:**
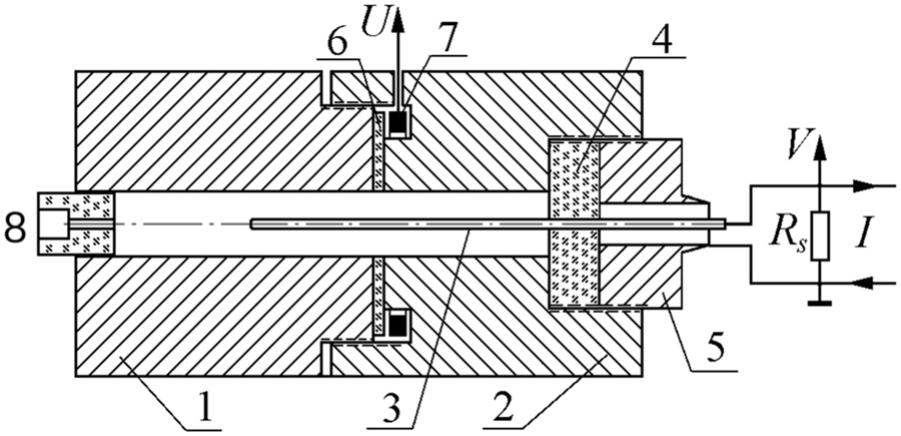
Experimental cell and feeding circuit.

The experimental procedures are described in detail in works^21,22^. Here, merely short description is presented. The explosive was pressed into a massive coaxial copper cell (Fig. 4). The outer electrode consisted of two parts 1 and 2 coupled with the thread connection. The central electrode 3 was mounted in the polymethyl methacrylate plug 4, supported by the hollow bolt 5. The charge diameter was *b* = 8 mm, and the diameter of the inner electrode was *c* = 2 mm. Toroidal coil 7 placed within the cavity formed in the outer electrode served as the conductivity gauge. Thin slit which connected the cavity with the coaxial was fixed by the dielectric layer 6 (0.3 mm thick for bulk density and 0.6 mm for pressed explosive).

BTF was pressed by equal portions whose masses corresponded to desired density. The length of each increment (5 mm) was controlled by special washers. Low density charges (1.1 g/cm^3^) were prepared by pouring 2.5 mm explosive portions into the coaxial, with slight compaction by the fixed weight. The experimental records demonstrate acceptable level of uniformity.

The charge was initiated through the axial channel in the PMMA stopper 8, filled with RDX. The constant feeding current *I* was supplied by the capacitor through the ballast resistor. The shunt *R*_*s*_ was connected in parallel with the cell. The feeding voltage *V*(*t*) and the gauge signal *U*(*t*) were measured. The conductivity at the slit plane is roughly proportional to the coil signal:

$$\sigma (x)=\frac{\mathrm{ln}(b/c)U(t)}{2\pi DMV(t)}\,\mathrm{.}$$

Here, *x* = *Dt* is the distance the detonation front moved after the slit at a time *t*, *D* is the detonation velocity, *V* is the voltage between the cell electrodes, *M* is the mutual inductance between the gauge contour and the surrounding cavity. Actually, the signal *U*(*t*) was corrected to eliminate the distortion caused by the coil inductance. The spatial resolution was estimated to be about a quarter of the width of a slit 6^21,22^. The instrumental error was estimated to be below 12%. Three shots were performed for each density of the BTF. Detailed description of the method and an animation illustrating the measurement can be found in supplementary materials.

**Figure 5:**
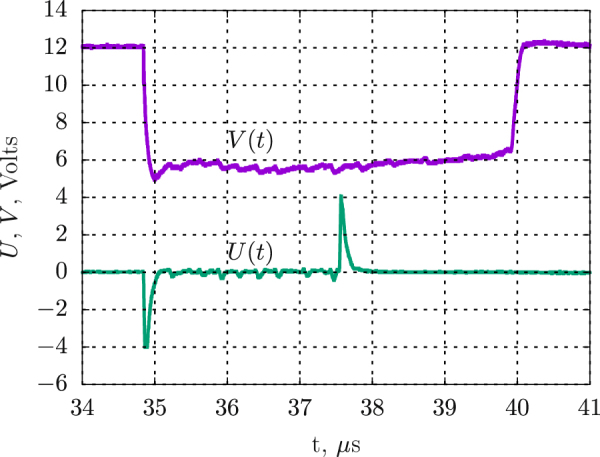
Cell voltage *V*(*t*) and gauge signal *U*(*t*) for BTF charge of 1.1 g/cm^3^ density.

Typical experimental record is shown in Fig. 5. The negative *U* peak at *t* = 34.8 *μ*s is induced by the starting cell current as the detonation wave reaches the central electrode. At the same moment, the voltage *V* falls down to the almost constant operating level (which indicates a good uniformity of the charge). The main signal *U* of positive polarity starts at about 37.5 *μ*s. At 40 *μ*s, the detonation wave reaches the end of the charge.

**Figure 6:**
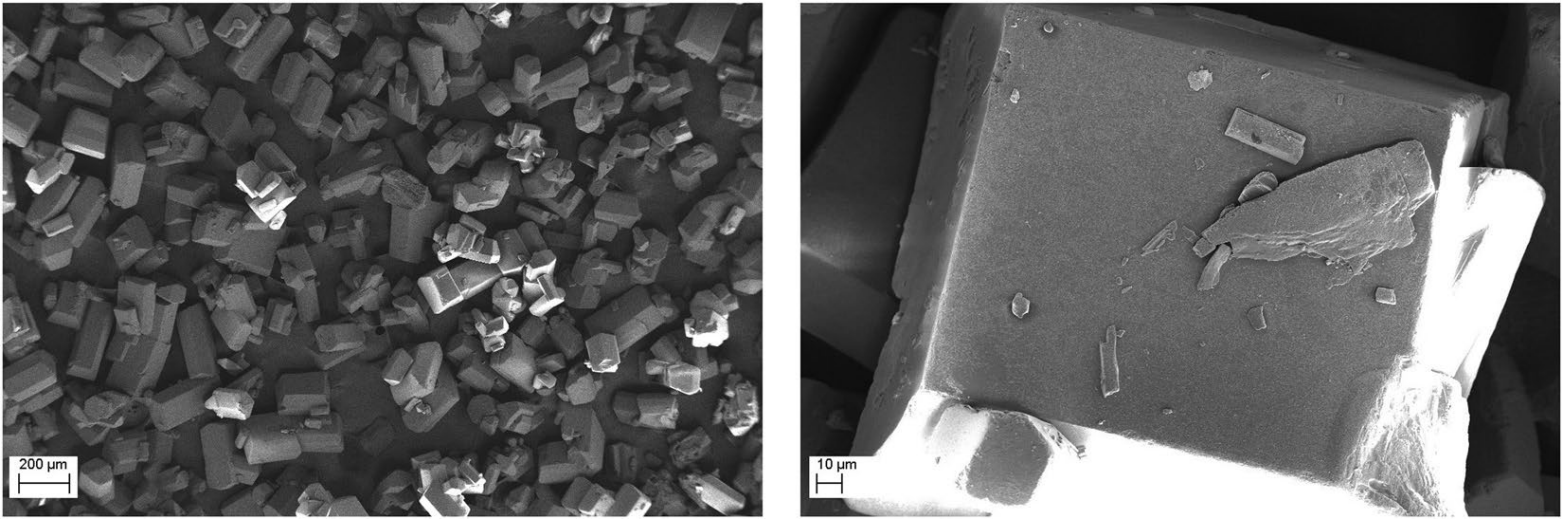
Micrographs of the BTF grains.

It was demonstrated earlier^23,24^ that the width of the conductivity peak at bulk initial density depends on the dispersity of the explosive. For the present experiments, characteristic grain size was around 150 *μ*m. The photographs of the BTF powder are shown in Fig. 6. Naturally, at higher density (after pressing) the initial grains are destroyed and fine grained structure is formed.

## Results

**Figure 7:**
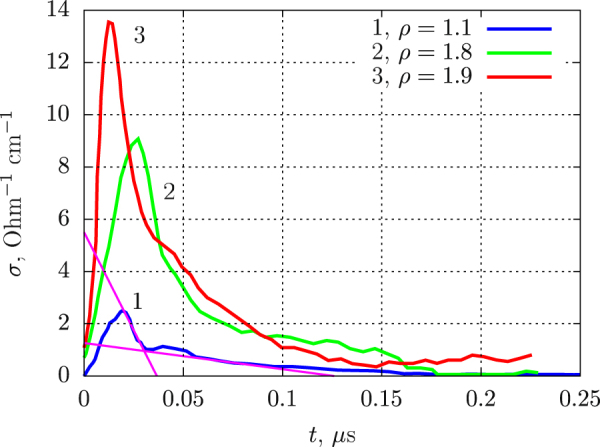
Electrical conductivity profiles in detonating BTF at different densities. For the density *ρ* = 1.1 g/cm^3^ a procedure defining the peak width is shown.

The effect of initial density on the conductivity is shown in Fig. 7. As for all other explosives peak conductivity increases with density. For all densities, the conductivity profile starts with the sharp peak, which presumably corresponds to the chemical reaction zone. After the peak, lower conductivity is observed in the Taylor wave. At *ρ* = 1.1 g/cm^3^, this residual conductivity is much lower than that in dense charges.

Experimental data are reported in the Table 1. Here, *ρ* is the initial density in g/cm^3^, *D* is the detonation velocity in km/s, *σ*_*max*_ is the peak conductivity in Ohm^−1^ cm^−1^, and *τ* is the duration of the peak in ns.

**Table 1:** Experimental data for BTF.

*ρ*	1.1	1.1	1.1	1.8	1.8	1.8	1.9	1.9	1.9
*D*	5.9	5.9	5.8	8.3	8.3	8.3	8.6	8.6	8.6
*σ*_*max*_	3.1	2.9	2.7	20.5	9.2	8.5	7.7	13.8	11
*τ*	35	77	89	28	48	47	59	38	60

The procedure of determining the peak duration (the same as that used in works^21,22^) is illustrated in Fig. 7 for *ρ* = 1.1 g/cm^3^. Note that for any given density, higher *σ*_*max*_ corresponds to shorter peak duration *τ*. Apparently, this correlation indicates distortion of the detonation front because of the charge non-uniformity. A slight inclination of the front, e.g. for 1 or 2 degrees, can extend the peak for 15–30 ns, with corresponding decrease of *σ*_*max*_. Thus, the minimal values of *τ* are the most reliable, so the reaction time in BTF can be estimated as 40 ns. At *ρ* = 1.8 g/cm^3^, maximal value of 20.5 Ohm^−1^ cm^−1^ was obtained. This value is similar to the data measured for other high explosives^22,23,25–28^.

It was found that in high density shots, the drop of the voltage *V*(*t*) after the contact of the detonation front with the central electrode was not monotonous. Instead, the transition to the new level of voltage was accompanied by oscillations of ≈6 ns peak to peak time, which died down after about 100 ns. These oscillations were generated in the low-impedance line (not shown in Fig. 4) which connected the coaxial cell to the shunt *R*_*s*_. The line was made of two bands of copper foil 20 cm long and 2 cm wide separated by 200 *μ*m plastic; it was used to protect the shunt from destruction (and hence the oscilloscope from dangerous voltage). The line capacitance was about 400 pF and inductances of both line and shunt were around 3 nH. To excite the oscillations, the measuring circuit should be closed fast enough (at least, faster than the period of oscillations). This means that the front of the conductivity profile in BTF is rather steep and the region of high conductivity is quite close to the detonation front (not more than 50 *μ*m distance). Other explosives studied earlier do not exhibit such behavior because of more gradual increase of the conductivity profile.

## Discussion

**Figure 8:**
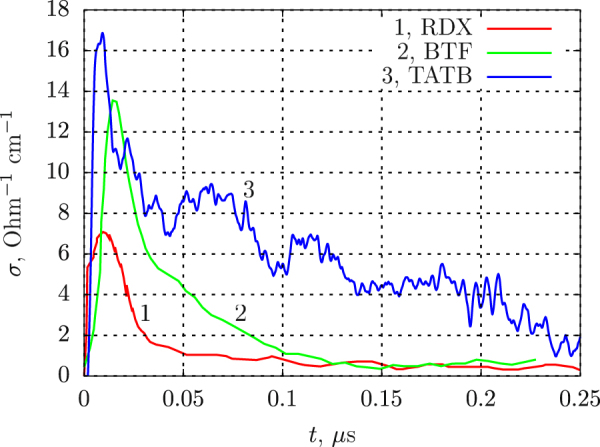
Electrical conductivity profiles in detonating dense explosives: RDX (*ρ* = 1.74 g/cm^3^), TATB (*ρ* = 1.8 g/cm^3^), BTF (*ρ* = 1.9 g/cm^3^).

Conductivity profiles in BTF are compared with those measured in RDX^21,29^ and TATB^30^ in Fig. 8. For all explosives, the profiles start with a distinct peak. It is reasonable to relate this peak to the reaction zone, as shown in works^22,28,31^. For instance, one can see that the conductivity peak in RDX is the narrowest while in TATB it is wider, in agreement with the expected difference in the reaction dynamics. On the contrary, the flow after the peak is considered to be basically equilibrium one, and the conductivity may depend on the chemical composition of the detonation products and on the thermodynamic parameters. The data presented in Fig. 8 call for discussion of the importance of various factors for the electrical conductivity.

### Role of temperature

The detonation temperature in TATB is 2750 K^3^, well below than that in BTF (4100 K according to the lowest estimate^5^). Nevertheless, peak conductivity in TATB is slightly higher than that in BTF. In RDX, the temperature value is intermediate (3600 K^3^) but the peak conductivity is lower than in both BTF and TATB. Thus, the temperature does not play a significant role, and processes such as thermal ionization of the detonation gases and thermal emission from the solid products are of secondary importance. It was shown earlier in work^32^ that the temperature effect on the conductivity is weak.

### Role of hydrogen

The electrical conductivity in detonation explosives was associated with the dissociation of water^1,2^. The water content in the detonation products of the CHNO explosives TNT, RDX, PETN, HMX, TATB is around 20%^3^. Since the conductivity of water shocked to the typical detonation pressure is ${\sigma }_{{H}_{2}O}\approx 10\,{{\rm{Ohm}}}^{-1}\,{{\rm{cm}}}^{-1}$^33,34^, this mechanism could explain the value of conductivity of several Ohm^−1^ cm^−1^ ^35^. A notable fact is that the conductivities of RDX and BTF outside the reaction zone (*t* > 0.1 *μ*s) are quite close (see Fig. 8). In contrast with RDX, BTF produces no water, thus, contribution of hydrogen ions in chemically equilibrated detonation products is minor at best. The same can be said about the reaction zone. The largest peak is in TATB, in BTF, the conductivity is slightly lower and the lowest value is found in RDX. This means that within the chemical peak, hydrogen and water do not prevail as well, and carbon is clearly much better candidate as the universal source of conductivity.

It should be noted that the authors of the “water-ionic” concept also studied the conductivity of hydrogen free explosive–liquid tetranitromethane CN_4_O_8_ ^36^. They found rather low conductivity within a reaction zone (~0.1 Ohm^−1^ cm^−1^) and much lower in the subsequent flow. To explain the results, dissociation of NO_2_ was suggested. Comparison with BTF case makes this idea doubtful because BTF clearly produces less NO_2_, but its conductivity, even at low density, is about 10 times higher. Again, the conductivity correlates rather with the carbon content, as discussed below.

### Role of carbon

First, let us discuss the post-reaction conductivity. The lower conductivity of BTF as compared with that of TATB may be considered as a confirmation of the contact model suggested by B. Hayes^18^ in which the grains of free carbon form conducting chains, or nets. There is less free carbon in the detonation products of BTF due to its better oxygen balance (−38.1% vs −55.8% in TATB). Moreover, the larger carbon particles produced in BTF detonation should be less inclined to form contacts and conducting structures. Smaller particles in TATB detonation products can form more contacts and hence provide higher electrical conductivity. The conductivity in RDX is still lower than in BTF, also in agreement with oxygen balance of RDX (−21.6%). So, the conductivity in detonation products is in good correlation with free carbon content and can be explained by the contact model.

All profiles exhibit the sharp conductivity peak. For all explosives discussed in the present work (RDX, BTF, TATB), the peak conductivity correlates with the mass content of carbon in the explosive molecule. For instance, TATB and BTF give close values of *σ*_*max*_, and their carbon mass fractions are almost identical: 0.279 and 0.286, respectively. For RDX, *σ*_*max*_ is about two times lower, at carbon mass fraction of 0.162. Role of the total molecular carbon content can be understood assuming that the carbon is released in the reaction zone in over-equilibrium quantities^37,38^. This view is based on the analysis of the experimental data on destruction of the shocked hydrocarbons. According to^39^, the molecules of hydrocarbons subjected to the strong shocks break out into condensed carbon and methane. In explosives, one may expect similar process (without methane in the case of BTF) followed by oxidation of these initial products. Thus, the quantity of carbon in the reaction zone may be higher than in detonation products. As a result, the conducting structures should be formed more effectively than in the subsequent flow. Besides, higher density in the reaction zone should further increase the conductivity. Preliminary considerations about the role of molecular carbon content were suggested in work^40^. A good correlation between the carbon mass fraction and the peak conductivity was shown in work^20^.

Thus, the results of experiments presented above strongly support the contact model of electrical conductivity of the carbon rich explosives. Formation of the conducting net from fine carbon particles can explain the conductivity both in the reaction zone and in the following flow.

## Conclusions

Despite the exotic chemical composition of BTF, its electrical conductivity distribution is generally similar to that measured in more common explosives (TATB, RDX, HMX, PETN). The conductivity profile starts with a sharp peak about 40 ns wide. The peak conductivity increases with density. The conductivity after the peak (*t* > 100 ns) is around 1 Ohm^−1^ cm^−1^ for high density explosive, and much lower for bulk density. Comparison with other explosives did not reveal any correlation between the temperature and the conductivity.

A sole special feature of BTF is a quite steep conductivity front. The rise time (no more than several ns) is beyond the resolution limit of our method (about 20 ns) and it manifested itself as rapidly damping noise at the start of the work of the experimental cell. As the absence of hydrogen did not produce other qualitative differences, dissociation of water or other compounds containing hydrogen into ions does not make a decisive contribution to the conductivity of common CHNO explosives.

The most probable mechanism of the conductivity in detonating BTF, as for other carbon rich explosives, is formation of conducting nets, or chains, from the carbon particles produced behind the detonation front.

## Electronic supplementary material

Supplementary Video

Supplementary Information
