# Author Correction: Electrical Conductivity Distribution in Detonating Benzotrifuroxane

**DOI:** 10.1038/s41598-018-29783-y

**Published:** 2018-08-01

**Authors:** Nataliya Satonkina, Alexander Ershov, Alexey Kashkarov, Anatoly Mikhaylov, Eduard Pruuel, Ivan Rubtsov, Ivan Spirin, Victoria Titova

**Affiliations:** 1 Lavrentyev Institute of Hydrodynamics SB RAS, pr. ac. Lavrentyeva, 15, Novosibirsk, 630090 Russia; 2 Novosibirsk State University, Pirogova str., 1, Novosibirsk, 630090 Russia; 3 Russian Federal Nuclear Center - VNIIEF, Sarov, Nizhny Novgorod region, Muzrukov Ave, 10, 607188 Russia

Correction to: *Scientific Reports* 10.1038/s41598-018-28028-2, published online 25 June 2018

This Article contains errors in Figure 8; the scale for the distribution of the electrical conductivity of the triaminotrinitrobenzene based explosive (TATB) is incorrect. The correct Figure 8 appears below as Figure 1.

**Figure 1:**
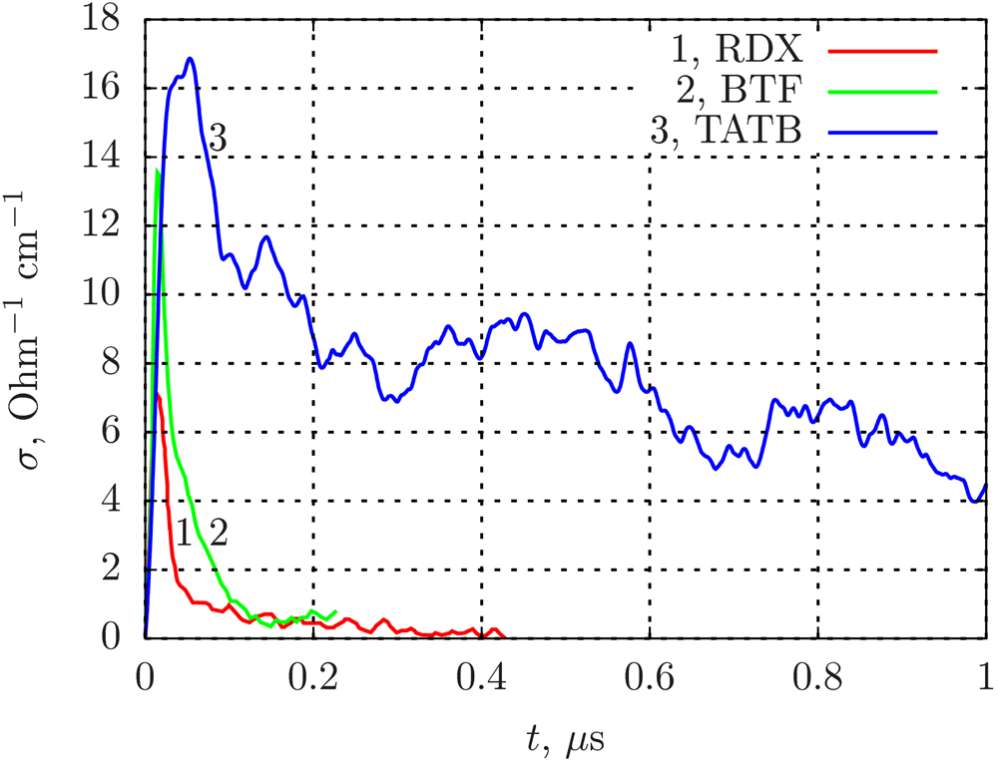
Electrical conductivity profiles in detonating dense explosives: RDX (*ρ* = 1.74 g/cm^3^), TATB (*ρ* = 1.8 g/cm^3^), BTF (*ρ* = 1.9 g/cm^3^).

