# Supplementary figures and images for: Electrical Conductivity Distribution in Detonating Benzotrifuroxane
Source: Sci Rep. 2018 Jun 25;8:9635. doi: 10.1038/s41598-018-28028-2 (PMC6018432; doi:10.1038/s41598-018-28028-2)

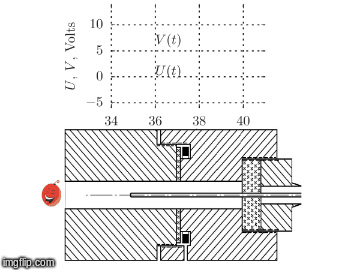

Supplement: Supplementary file 1 — Supplementary Video [file 41598_2018_28028_MOESM1_ESM.gif]
